# Supplementary material for: Heparin-based hydrogel scaffolding alters the transcriptomic profile and increases the chemoresistance of MDA-MB-231 triple-negative breast cancer cells
Source: Biomater Sci. 2020 Feb 13;8(10):2786–96. doi: 10.1039/c9bm01481k (PMC7497406; doi:10.1039/c9bm01481k)
Supplement: Supplementary file 2 [file BM-008-C9BM01481K-s002.zip › Supplementary File 4/HP-BhydrogelvControl/Pathways/my_analysis.Gsea.1545201079907/HALLMARK_FATTY_ACID_METABOLISM.html]

Details for gene set HALLMARK\_FATTY\_ACID\_METABOLISM[GSEA]

|  || Dataset | expr.class.cls#HYD\_versus\_CONTROL.class.cls#HYD\_versus\_CONTROL\_repos |
| Phenotype | class.cls#HYD\_versus\_CONTROL\_repos |
| Upregulated in class | CONTROL |
| GeneSet | HALLMARK\_FATTY\_ACID\_METABOLISM |
| Enrichment Score (ES) | -0.46442565 |
| Normalized Enrichment Score (NES) | -2.1162934 |
| Nominal p-value | 0.0 |
| FDR q-value | 0.0 |
| FWER p-Value | 0.0 |
Table: GSEA Results Summary

  

Fig 1: Enrichment plot: HALLMARK\_FATTY\_ACID\_METABOLISM      
 Profile of the Running ES Score & Positions of GeneSet Members on the Rank Ordered List

  

| PROBE | DESCRIPTION (from dataset) | GENE SYMBOL | GENE\_TITLE | RANK IN GENE LIST | RANK METRIC SCORE | RUNNING ES | CORE ENRICHMENT || 1 | MLYCD | na |  |  | 231 | 2.846 | 0.0131 | No |
| 2 | D2HGDH | na |  |  | 292 | 2.691 | 0.0337 | No |
| 3 | FASN | na |  |  | 563 | 2.266 | 0.0396 | No |
| 4 | SLC22A5 | na |  |  | 826 | 2.011 | 0.0437 | No |
| 5 | GCDH | na |  |  | 1111 | 1.792 | 0.0447 | No |
| 6 | ENO3 | na |  |  | 1277 | 1.702 | 0.0511 | No |
| 7 | ACADVL | na |  |  | 1828 | 1.430 | 0.0349 | No |
| 8 | AOC3 | na |  |  | 2154 | 1.295 | 0.0293 | No |
| 9 | ACAA1 | na |  |  | 3257 | 0.975 | -0.0199 | No |
| 10 | CPT1A | na |  |  | 3541 | 0.908 | -0.0267 | No |
| 11 | NCAPH2 | na |  |  | 3807 | 0.850 | -0.0331 | No |
| 12 | LTC4S | na |  |  | 3921 | 0.828 | -0.0317 | No |
| 13 | ACOT2 | na |  |  | 4077 | 0.794 | -0.0328 | No |
| 14 | CEL | na |  |  | 4492 | 0.719 | -0.0481 | No |
| 15 | ECI1 | na |  |  | 4503 | 0.716 | -0.0423 | No |
| 16 | CYP1A1 | na |  |  | 4586 | 0.695 | -0.0405 | No |
| 17 | AADAT | na |  |  | 4594 | 0.693 | -0.0347 | No |
| 18 | IL4I1 | na |  |  | 4920 | 0.638 | -0.0461 | No |
| 19 | ACSS1 | na |  |  | 5797 | 0.514 | -0.0875 | No |
| 20 | AQP7 | na |  |  | 6056 | 0.479 | -0.0968 | No |
| 21 | CPT2 | na |  |  | 6409 | 0.429 | -0.1114 | No |
| 22 | PTPRG | na |  |  | 6425 | 0.427 | -0.1084 | No |
| 23 | MGLL | na |  |  | 7102 | 0.348 | -0.1408 | No |
| 24 | CPOX | na |  |  | 7197 | 0.339 | -0.1427 | No |
| 25 | AUH | na |  |  | 7536 | 0.302 | -0.1578 | No |
| 26 | GPD1 | na |  |  | 7544 | 0.300 | -0.1555 | No |
| 27 | IDH3G | na |  |  | 7740 | 0.282 | -0.1632 | No |
| 28 | ALDH3A1 | na |  |  | 8629 | 0.188 | -0.2081 | No |
| 29 | ACADS | na |  |  | 8847 | 0.159 | -0.2181 | No |
| 30 | ODC1 | na |  |  | 8852 | 0.159 | -0.2169 | No |
| 31 | PPARA | na |  |  | 9189 | 0.127 | -0.2334 | No |
| 32 | ME1 | na |  |  | 9632 | 0.084 | -0.2558 | No |
| 33 | ECH1 | na |  |  | 9762 | 0.069 | -0.2620 | No |
| 34 | NTHL1 | na |  |  | 9986 | 0.042 | -0.2733 | No |
| 35 | EPHX1 | na |  |  | 10016 | 0.038 | -0.2745 | No |
| 36 | SDHA | na |  |  | 10187 | 0.016 | -0.2833 | No |
| 37 | RETSAT | na |  |  | 10420 | 0.005 | -0.2954 | No |
| 38 | GRHPR | na |  |  | 10557 | -0.011 | -0.3024 | No |
| 39 | HSD17B10 | na |  |  | 10723 | -0.032 | -0.3108 | No |
| 40 | TP53INP2 | na |  |  | 10838 | -0.044 | -0.3164 | No |
| 41 | VNN1 | na |  |  | 10879 | -0.050 | -0.3180 | No |
| 42 | ENO2 | na |  |  | 11078 | -0.075 | -0.3278 | No |
| 43 | CRAT | na |  |  | 11449 | -0.107 | -0.3462 | No |
| 44 | GSTZ1 | na |  |  | 11501 | -0.111 | -0.3479 | No |
| 45 | HIBCH | na |  |  | 11525 | -0.116 | -0.3481 | No |
| 46 | ALDH3A2 | na |  |  | 11689 | -0.137 | -0.3554 | No |
| 47 | DHCR24 | na |  |  | 11725 | -0.142 | -0.3560 | No |
| 48 | G0S2 | na |  |  | 11856 | -0.159 | -0.3614 | No |
| 49 | HMGCS1 | na |  |  | 12129 | -0.190 | -0.3740 | No |
| 50 | ACOT8 | na |  |  | 12300 | -0.213 | -0.3810 | No |
| 51 | ACAT2 | na |  |  | 12500 | -0.224 | -0.3895 | No |
| 52 | ACO2 | na |  |  | 12768 | -0.255 | -0.4012 | No |
| 53 | PCBD1 | na |  |  | 13000 | -0.283 | -0.4109 | No |
| 54 | DLST | na |  |  | 13510 | -0.354 | -0.4344 | No |
| 55 | ACSL5 | na |  |  | 13564 | -0.364 | -0.4340 | No |
| 56 | ACADL | na |  |  | 13643 | -0.378 | -0.4347 | No |
| 57 | ALAD | na |  |  | 13731 | -0.393 | -0.4358 | No |
| 58 | METAP1 | na |  |  | 13774 | -0.398 | -0.4345 | No |
| 59 | ACSL4 | na |  |  | 13801 | -0.403 | -0.4323 | No |
| 60 | BPHL | na |  |  | 13805 | -0.404 | -0.4289 | No |
| 61 | REEP6 | na |  |  | 13830 | -0.411 | -0.4265 | No |
| 62 | CBR3 | na |  |  | 13871 | -0.414 | -0.4249 | No |
| 63 | HMGCL | na |  |  | 14016 | -0.419 | -0.4288 | No |
| 64 | CCDC58 | na |  |  | 14379 | -0.474 | -0.4436 | No |
| 65 | ECI2 | na |  |  | 14523 | -0.495 | -0.4467 | No |
| 66 | ACOX1 | na |  |  | 14591 | -0.504 | -0.4457 | No |
| 67 | ACSL1 | na |  |  | 14604 | -0.507 | -0.4419 | No |
| 68 | NBN | na |  |  | 14611 | -0.508 | -0.4377 | No |
| 69 | HADH | na |  |  | 14717 | -0.526 | -0.4385 | No |
| 70 | IDH3B | na |  |  | 14744 | -0.530 | -0.4352 | No |
| 71 | ERP29 | na |  |  | 15302 | -0.618 | -0.4590 | Yes |
| 72 | ALDOA | na |  |  | 15303 | -0.618 | -0.4535 | Yes |
| 73 | PDHA1 | na |  |  | 15305 | -0.618 | -0.4481 | Yes |
| 74 | HSPH1 | na |  |  | 15335 | -0.623 | -0.4441 | Yes |
| 75 | UROS | na |  |  | 15403 | -0.635 | -0.4420 | Yes |
| 76 | PTS | na |  |  | 15494 | -0.649 | -0.4410 | Yes |
| 77 | MCEE | na |  |  | 15584 | -0.666 | -0.4397 | Yes |
| 78 | UBE2L6 | na |  |  | 15591 | -0.666 | -0.4342 | Yes |
| 79 | H2AFZ | na |  |  | 15730 | -0.688 | -0.4353 | Yes |
| 80 | GABARAPL1 | na |  |  | 15826 | -0.709 | -0.4340 | Yes |
| 81 | SMS | na |  |  | 15905 | -0.723 | -0.4317 | Yes |
| 82 | GPD2 | na |  |  | 16014 | -0.750 | -0.4307 | Yes |
| 83 | CBR1 | na |  |  | 16064 | -0.762 | -0.4266 | Yes |
| 84 | YWHAH | na |  |  | 16078 | -0.765 | -0.4205 | Yes |
| 85 | ECHS1 | na |  |  | 16197 | -0.787 | -0.4197 | Yes |
| 86 | GLUL | na |  |  | 16401 | -0.832 | -0.4230 | Yes |
| 87 | RDH11 | na |  |  | 16414 | -0.835 | -0.4162 | Yes |
| 88 | DLD | na |  |  | 16423 | -0.837 | -0.4092 | Yes |
| 89 | SUCLG2 | na |  |  | 16425 | -0.837 | -0.4019 | Yes |
| 90 | IDI1 | na |  |  | 16426 | -0.838 | -0.3945 | Yes |
| 91 | ACADM | na |  |  | 16495 | -0.853 | -0.3905 | Yes |
| 92 | DECR1 | na |  |  | 16501 | -0.855 | -0.3832 | Yes |
| 93 | ACSM3 | na |  |  | 16572 | -0.869 | -0.3791 | Yes |
| 94 | BCKDHB | na |  |  | 16597 | -0.871 | -0.3727 | Yes |
| 95 | HSD17B7 | na |  |  | 16661 | -0.888 | -0.3681 | Yes |
| 96 | ADIPOR2 | na |  |  | 16920 | -0.962 | -0.3732 | Yes |
| 97 | MDH1 | na |  |  | 16955 | -0.971 | -0.3663 | Yes |
| 98 | HADHB | na |  |  | 17247 | -1.066 | -0.3722 | Yes |
| 99 | NSDHL | na |  |  | 17265 | -1.072 | -0.3636 | Yes |
| 100 | BLVRA | na |  |  | 17422 | -1.122 | -0.3618 | Yes |
| 101 | HSD17B4 | na |  |  | 17589 | -1.191 | -0.3600 | Yes |
| 102 | HSDL2 | na |  |  | 17594 | -1.193 | -0.3496 | Yes |
| 103 | MDH2 | na |  |  | 17620 | -1.207 | -0.3403 | Yes |
| 104 | APEX1 | na |  |  | 17668 | -1.228 | -0.3319 | Yes |
| 105 | BMPR1B | na |  |  | 17678 | -1.234 | -0.3214 | Yes |
| 106 | PSME1 | na |  |  | 17774 | -1.276 | -0.3151 | Yes |
| 107 | HCCS | na |  |  | 17784 | -1.280 | -0.3043 | Yes |
| 108 | EHHADH | na |  |  | 17881 | -1.324 | -0.2976 | Yes |
| 109 | SDHC | na |  |  | 17944 | -1.344 | -0.2889 | Yes |
| 110 | ETFDH | na |  |  | 17960 | -1.356 | -0.2777 | Yes |
| 111 | ALDH9A1 | na |  |  | 18095 | -1.437 | -0.2720 | Yes |
| 112 | ADSL | na |  |  | 18103 | -1.441 | -0.2596 | Yes |
| 113 | LGALS1 | na |  |  | 18128 | -1.458 | -0.2480 | Yes |
| 114 | IDH1 | na |  |  | 18161 | -1.475 | -0.2366 | Yes |
| 115 | RAP1GDS1 | na |  |  | 18202 | -1.501 | -0.2254 | Yes |
| 116 | SDHD | na |  |  | 18206 | -1.504 | -0.2123 | Yes |
| 117 | MAOA | na |  |  | 18217 | -1.509 | -0.1995 | Yes |
| 118 | SUCLA2 | na |  |  | 18223 | -1.511 | -0.1863 | Yes |
| 119 | FH | na |  |  | 18267 | -1.540 | -0.1750 | Yes |
| 120 | UROD | na |  |  | 18338 | -1.593 | -0.1645 | Yes |
| 121 | UGDH | na |  |  | 18399 | -1.641 | -0.1532 | Yes |
| 122 | PDHB | na |  |  | 18418 | -1.650 | -0.1395 | Yes |
| 123 | ELOVL5 | na |  |  | 18504 | -1.707 | -0.1289 | Yes |
| 124 | HSD17B11 | na |  |  | 18720 | -1.913 | -0.1232 | Yes |
| 125 | ACAA2 | na |  |  | 18837 | -2.070 | -0.1110 | Yes |
| 126 | PRDX6 | na |  |  | 18878 | -2.137 | -0.0942 | Yes |
| 127 | CRYZ | na |  |  | 18909 | -2.177 | -0.0765 | Yes |
| 128 | SUCLG1 | na |  |  | 19027 | -2.431 | -0.0611 | Yes |
| 129 | SERINC1 | na |  |  | 19065 | -2.591 | -0.0401 | Yes |
| 130 | OSTC | na |  |  | 19068 | -2.600 | -0.0172 | Yes |
| 131 | S100A10 | na |  |  | 19095 | -2.708 | 0.0054 | Yes |
Table: GSEA details [plain text format]

  

Fig 2: HALLMARK\_FATTY\_ACID\_METABOLISM      
 Blue-Pink O' Gram in the Space of the Analyzed GeneSet

  

Fig 3: HALLMARK\_FATTY\_ACID\_METABOLISM: Random ES distribution      
 Gene set null distribution of ES for **HALLMARK\_FATTY\_ACID\_METABOLISM**
